# Sweet Taste Receptor Expressed in Pancreatic β-Cells Activates the Calcium and Cyclic AMP Signaling Systems and Stimulates Insulin Secretion

**DOI:** 10.1371/journal.pone.0005106

**Published:** 2009-04-08

**Authors:** Yuko Nakagawa, Masahiro Nagasawa, Satoko Yamada, Akemi Hara, Hideo Mogami, Viacheslav O. Nikolaev, Martin J. Lohse, Noriatsu Shigemura, Yuzo Ninomiya, Itaru Kojima

**Affiliations:** 1 Institute for Molecular and Cellular Regulation, Gunma University, Maebashi, Japan; 2 Department of Physiology, Hamamatsu Medical School, Hamamatsu, Japan; 3 Institute of Pharmacology and Toxicology, University of Wurzburg, Wurzburg, Germany; 4 Section of Oral Neuroscience, Graduate School of Dental Science, Kyushu University, Fukuoka, Japan; University of Bremen, Germany

## Abstract

**Background:**

Sweet taste receptor is expressed in the taste buds and enteroendocrine cells acting as a sugar sensor. We investigated the expression and function of the sweet taste receptor in MIN6 cells and mouse islets.

**Methodology/Principal Findings:**

The expression of the sweet taste receptor was determined by RT–PCR and immunohistochemistry. Changes in cytoplasmic Ca^2+^ ([Ca^2+^]_c_) and cAMP ([cAMP]_c_) were monitored in MIN6 cells using fura-2 and Epac1-camps. Activation of protein kinase C was monitored by measuring translocation of MARCKS-GFP. Insulin was measured by radioimmunoassay. mRNA for T1R2, T1R3, and gustducin was expressed in MIN6 cells. In these cells, artificial sweeteners such as sucralose, succharin, and acesulfame-K increased insulin secretion and augmented secretion induced by glucose. Sucralose increased biphasic increase in [Ca^2+^]_c_. The second sustained phase was blocked by removal of extracellular calcium and addition of nifedipine. An inhibitor of inositol(1, 4, 5)-trisphophate receptor, 2-aminoethoxydiphenyl borate, blocked both phases of [Ca^2+^]_c_ response. The effect of sucralose on [Ca^2+^]_c_ was inhibited by gurmarin, an inhibitor of the sweet taste receptor, but not affected by a G_q_ inhibitor. Sucralose also induced sustained elevation of [cAMP]_c_, which was only partially inhibited by removal of extracellular calcium and nifedipine. Finally, mouse islets expressed T1R2 and T1R3, and artificial sweeteners stimulated insulin secretion.

**Conclusions:**

Sweet taste receptor is expressed in β-cells, and activation of this receptor induces insulin secretion by Ca^2+^ and cAMP-dependent mechanisms.

## Introduction

Molecular identification of the sweet taste receptor has provided new and precise insights into our understanding of the taste sensation [1]–[4]. The sweet taste receptor is a heterodimer of T1R2 and T1R3, both of which belong to a subclass of G-protein-coupled receptors resembling metabotropic glutamate receptor (mGluR), calcium-sensing receptor and pheromone receptors (V2R). Members of this subclass have large extracellular amino-terminal domains which bind most of the ligands to this region. Based on structural similarity with mGluR1, binding of ligands is thought to stabilize the active form of the sweet receptor by binding them within the cleft. Indeed, the sweet taste receptor is activated by various types of sweet substances including glucose, sucrose, fructose, artificial sweeteners including saccharin and acesulfame-K, and even proteins such as monellin and thaumatin [5], [6]. Most of them are small molecules but some are much larger in size. It is thought that various types of sweeteners bind to different portions of the receptor, stabilize by different manners, and activate the βγ subunit of the trimeric G protein, which subsequently activates phospholipase C-β (PLC-β) (5, 6).

In addition to the taste cells in the tongue, the sweet taste receptor is also expressed in intestinal epithelial cells, in particular, in enteroendocrine cells [7]. Margolskee, et al. [8] recently showed that the sweet taste receptor expressed in enteroendocrine cells regulates the expression of sodium-dependent glucose transporter-1 (SGLT1), which is expressed in enterocytes. Activators of the sweet taste receptor including dietary sugar and artificial sweeteners activate the sweet taste receptor expressed in enteroendocrine cells and induce secretion of glucagon-like peptide-1 and glucose-dependent insulinotropic peptide, both of which stimulate the expression of SGLT1 in enterocytes. These observations clearly demonstrate that the sweet taste receptor functions as a sugar sensor in tissues other than taste buds in the tongue.

In this regard, pancreatic β-cells are originated from endoderm and resemble enteroendocrine cells in many respects [9], [10]. More importantly, these cells respond to fuels, especially sugars, including glucose and secrete insulin, a primary regulator of the glucose metabolism in the body. It is now generally accepted that the glucose-sensing machinery in β-cells is dependent on glucose metabolism [11], and molecules such as glucokinase and ATP-sensitive potassium channel are important for glucose sensing. Nevertheless, given that the sweet taste receptor is a key molecule in sugar sensing, it is interesting to address whether or not the sweet taste receptor is expressed in β cells. In the present study, we examined whether or not the sweet taste receptor is expressed in pancreatic β-cells. We also investigated the function of this receptor using MIN6 cells.

## Results

### Expression of the Sweet Taste Receptor in MIN6 Cells

**Figure 1:**
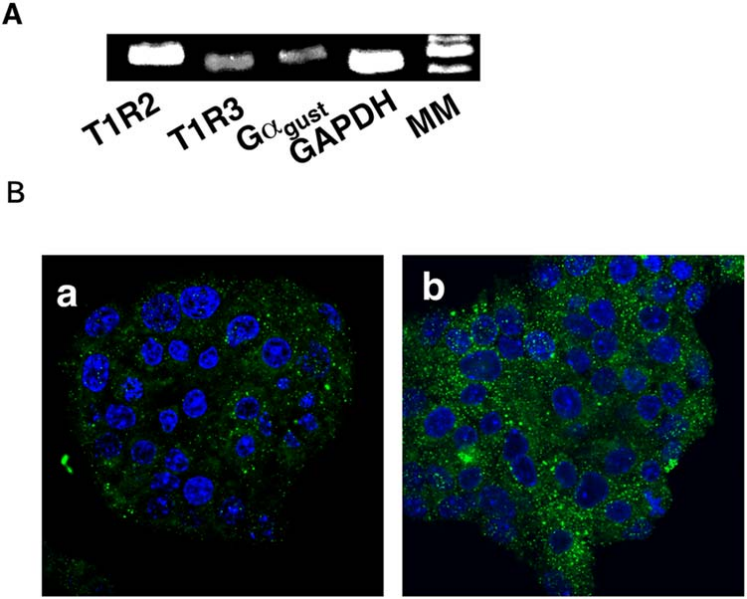
Expression of the sweet taste receptor in MIN6 cells. (A) Expression of mRNA for T1R2, T1R3 and α subunit of gustducin (Gα_gust_) in MIN6 cells was measured by RT-PCR. MM: molecular markers. The result is a representative of three experiments. (B) MIN6 cells were stained by anti-T1R2 (a) and anti-T1R3 (b) antibodies.

We first examined whether or not the sweet taste receptor is expressed in MIN6 cells. As shown in Figure 1A, mRNA for T1R2 and T1R3 was detected by RT-PCR. In addition, mRNA for gustducin was also expressed in MIN6 cells. We then examined the expression of the sweet taste receptor by immunohistochemistry. Immunoreactivities of T1R2 and T1R3 were detected in MIN6 cells (Figure 1B). T1R3 signal was stronger and punctated.

### Effect of Artificial Sweeteners on Insulin Secretion in MIN6 Cells

**Figure 2:**
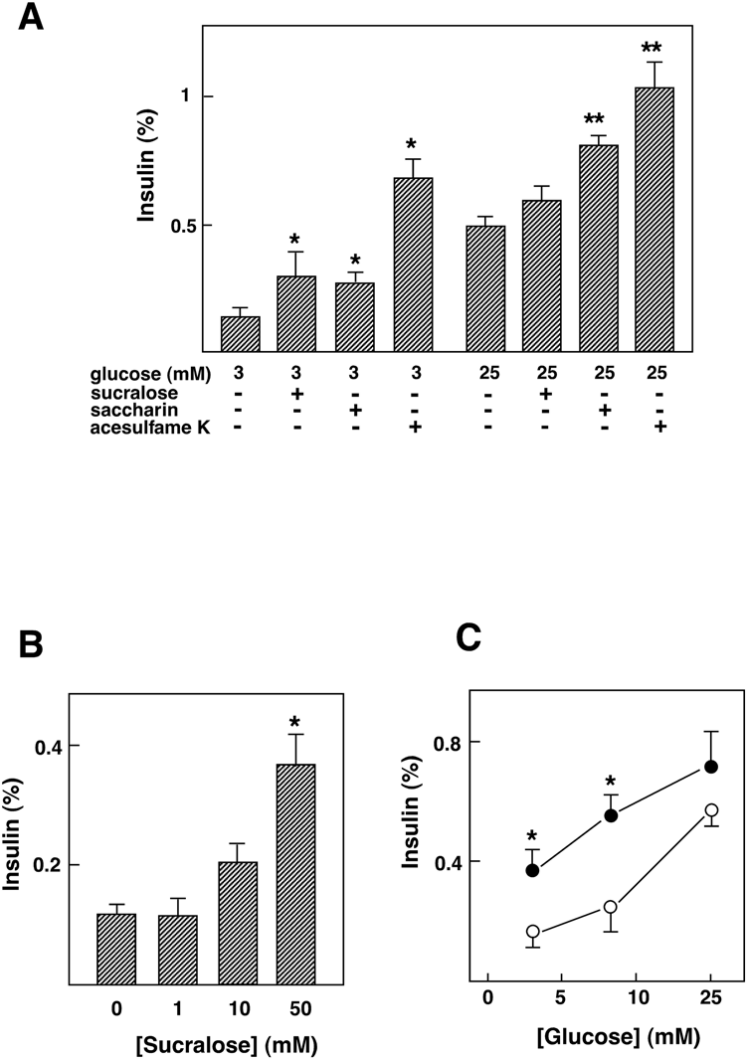
Effect of artificial sweeteners on insulin secretion in MIN6 cells. (A) MIN6 cells were incubated for 60 min with or without 50 mM saccharin, 50 mM sucralose or 50 mM acesulfame-K in the presence of 3 or 25 mM glucose, and insulin secretion was measured. Values are expressed as means±S.E. for four experiments. *: P<0.05 vs 3 mM glucose, **: P<0.05 vs 25 mM glucose. Note that 50 mM mannitol did not affect insulin secretion. (B) MIN6 cells were incubated for 60 min with 0, 1, 10 or 50 mM sucralose in the presence of 3 mM glucose, and insulin secretion was measured. Values are expressed as mean±S. E. for four experiments. *: P<0.05 vs 3 mM glucose alone. (C) MIN6 cells were incubated for 60 min with 3, 8.3 or 25 mM glucose in the presence (•) and absence (○) of 50 mM sucralose. Values are expressed as mean±S. E. for four experiments. *: P<0.05 vs without sucralose.

To determine the function of the sweet taste receptor expressed in MIN6 cells, we examined whether artificial sweeteners affected insulin secretion. As shown in Figure 2A, sucralose stimulated insulin secretion in the presence of a low concentration of glucose. Likewise, saccharin stimulated insulin secretion and acesulfame-K was much more potent. Note that 50 mM mannitol did not affect insulin secretion (data not shown). Saccharin and acesulfame-K also increased insulin secretion induced by a high concentration of glucose. Figure 2B depicts the dose-response relationship of the sucralose effect. The effect of sucralose was evident at a concentration of 50 mM. As shown in Figure 2C, sucralose augmented glucose-induced insulin secretion. Hence, agonists of the sweet taste receptor in the taste buds of the tongue were able to induce insulin secretion from MIN6 cells.

### Changes in Cytoplasmic Ca^2+^ and cAMP Concentrations

**Figure 3:**
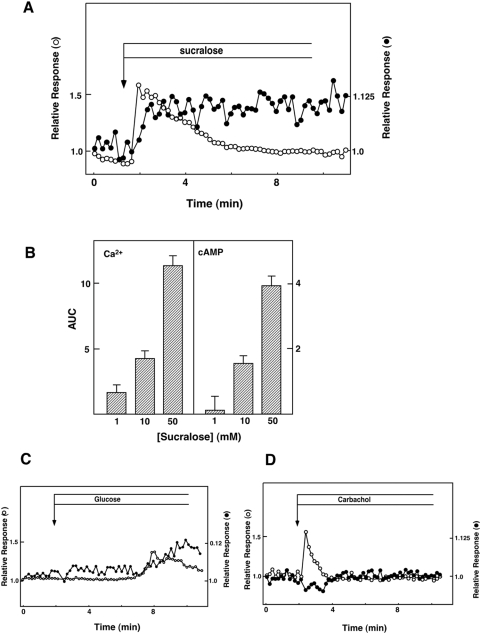
Effect of sucralose on [Ca^2+^]_c_ and [cAMP]_c_ in MIN6 cells. (A) MIN6 cells expressing Epac1-camps were loaded with fura-2, and changes in [Ca^2+^]_c_ (○) and [cAMP]_c_ (•) were monitored. Cells were stimulated by 50 mM sucralose. Note that 50 mM mannitol did not affect [Ca^2+^]_c_ or [cAMP]_c_ indicating that the effect of sucralose was not simply due to changes in osmolarity. (B) Dose-response relationship for the effect of sucralose. Cells were stimulated with various concentrations of sucralose, and the area under the curve (AUC) for [Ca^2+^]_c_ and [cAMP]_c_ was calculated. Values are the mean±S.E. for five experiments. (C) Epac1-camps-expressing cells loaded with fura-2 were stimulated with 25 mM glucose, and changes in [Ca^2+^]_c_ (○) and [cAMP]_c_ (•) were monitored. (D) Cells were stimulated with 50 µM carbachol and changes in [Ca^2+^]_c_ (○) and [cAMP]_c_ (•) were monitored.

**Figure 4:**
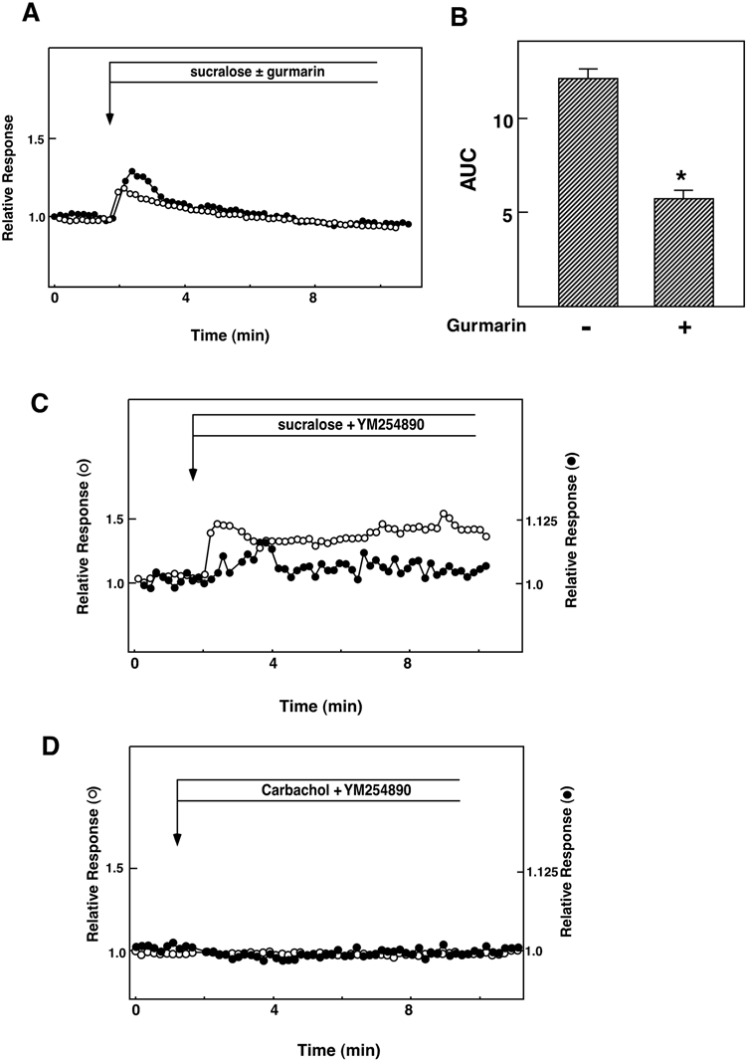
Involvement of the sweet taste receptor in the action of sucralose. (A): Epac1-camps-expressing MIN6 cells loaded with fura-2 were incubated with (○) or without (•) 3 µg/ml gurmarin for 10 min and then stimulated with 50 mM sucralose. Changes in [Ca^2+^]_c_ were monitored. (B) Quantitative analysis of the effect of gurmarin. Cells were stimulated by 50 mM sucralose in the presence and absence of gurmarin and the AUC was calculated. Values are the mean±S.E. for four experiments. *: p<0.05 vs none. (C) Epac1-camps-expressing MIN6 cells loaded with fura-2 were preincubated with 10 µM YM254890 for 10 min and then stimulated with 50 mM sucralose in the presence of YM254890. Changes in [Ca^2+^]_c_ (○) and [cAMP]_c_ (•) were monitored. (D) Epac1-camps-expressing MIN6 cells loaded with fura-2 were preincubated with YM254890 for 10 min and then stimulated with 50 µM carbachol in the presence (○) and absence (•) of YM254890.

**Figure 5:**
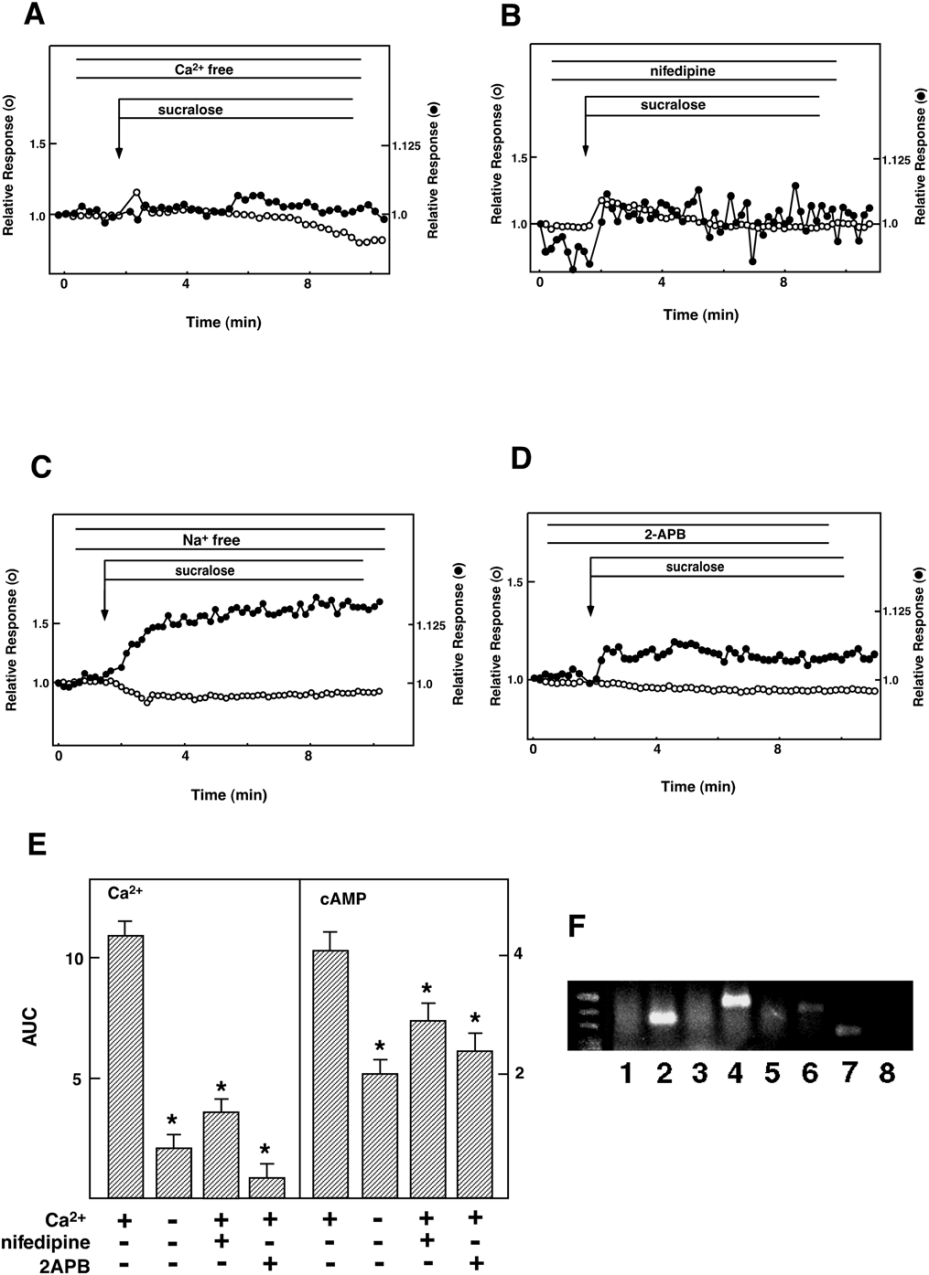
Role of calcium entry in the action of sucralose on [Ca^2+^]_c_. (A) Epac1-camps-expressing MIN6 cells loaded with fura-2 were stimulated by 50 mM sucralose in calcium-free HBSS, and changes in [Ca^2+^]_c_ (○) and [cAMP]_c_ (•) were monitored. (B) Epac1-camps-expressing MIN6 cells loaded with fura-2 were stimulated by 50 mM sucralose in the presence of 1 µM nifedipine. (C) Epac1-camps-expressing MIN6 cells loaded with fura-2 were incubated in Na-free HBSS and stimulated with 50 mM sucralose and changes in [Ca^2+^]_c_ (○) and [cAMP]_c_ (•) were measured. (D) Epac1-camps-expressing MIN6 cells loaded with fura-2 were stimulated with 50 mM sucralose in the presence of 200 µM 2APB and changes in [Ca^2+^]_c_ (○) and [cAMP]_c_ (•) were measured. (E) Quantitative analysis of the above data. *: P<0.05. (F) Expression of TRPM channels in MIN6 cells. Expression of various types of TRPM channels was measured by RT-PCR using mRNA obtained from MIN6 cells. 1, 2, 3, 4, 5, 6, 7, and 8 stand for TRPM1, TRPM2, TRPM3, TRPM4, TRPM5, TRPM6, TRPM7, TRPM8, respectively.

To investigate the intracellular signaling system activated by the sweet taste receptor, we measured the effects of sucralose on [Ca^2+^]_c_ and [cAMP]_c_. We monitored [Ca^2+^]_c_ and [cAMP]_c_ simultaneously using fura-2 and Epac1-camps. As shown in Figure 3A, sucralose induced increases in both [Ca^2+^]_c_ and [cAMP]_c_. Sucralose actually induced biphasic elevation of [Ca^2+^]_c_. The initial rapid peak was followed by a gradual decrease in [Ca^2+^]_c_, and [Ca^2+^]_c_ remained slightly elevated for a long period. The effect of sucralose was dose-dependent and detected at a concentration of 10 mM (Figure 3B). Note that the effect of sucralose on [Ca^2+^]_c_ was not affected by diazoxide, an opener of the ATP-sensitive potassium channel (data not shown). The patterns of changes in [Ca^2+^]_c_ and [cAMP]_c_ were quite different from that induced by the high concentration of glucose (Figure 3C). As depicted, 25 mM glucose increased [Ca^2+^]_c_ after a lag period of 2 to 5 min, which was accompanied by an elevation of [cAMP]_c_. The effect of sucralose was also different from that induced by carbachol, a muscarinic agonist which activates PLC-β. As depicted in Figure 3D, carbachol induced a transient increase in [Ca^2+^]_c_. In contrast, [cAMP]_c_ was reduced by carbachol. We then examined the involvement of the sweet taste receptor in the action of sucralose. As shown in Figure 4A, 3 µg/ml gurmarin, an inhibitor of the sweet taste receptor in the taste buds [12] attenuated sucralose-induced elevation of [Ca^2+^]_c_. Quantitatively, the effect of gurmarin was statistically significant (Figure 4B). Higher concentration of gurmarin did not further inhibit the sucralose action. Gurmarin did not affect [cAMP]_c_ response to sucralose (data not shown). We then examined the involvement of G_q/11_ in the action of sucralose. As shown in Figure 4C, YM254890, an inhibitor of G_q/11_ [13], barely inhibited the elevation of [Ca^2+^]_c_ and [cAMP]_c_ induced by sucralose. In contrast, the effects of carbachol on [Ca^2+^]_c_ and [cAMP]_c_ were completely blocked by YM254890 (Figure 4D). We then examined the dependency of [Ca^2+^]_c_ and [cAMP]_c_ responses on extracellular calcium. As shown in Figure 5A, removal of extracellular calcium markedly reduced the [Ca^2+^]_c_ response to sucralose. In the absence of extracellular calcium, the effect of sucralose on [Ca^2+^]_c_ was small and only transient. Furthermore, the effect of sucralose on [cAMP]_c_ was attenuated. Similarly, [Ca^2+^]_c_ response to sucralose was markedly inhibited by nifedipine, an inhibitor of the L-type voltage-dependent calcium channel. Again, nifedipine also reduced elevation of [cAMP]_c_ induced by sucralose (Figure 5B). When extracellular sodium was removed, the effect of sucralose on [Ca^2+^]_c_ was greatly reduced (Figure 5C). We also examined the effect of an inhibitor of inositol(1,4,5)-trisphosphate (Ins-P_3_) receptor 2-aminoethoxydiphenyl borate (2APB) [14]. As shown in Figure 5D, 2APB nearly completely blocked sucralose-induced elevation of [Ca^2+^]_c_. Both initial and sustained phases of the [Ca^2+^]_c_ response were blocked. In this condition, [cAMP]_c_ response was also reduced but not blocked completely. Quantitative analyses of the effects of these agents are summarized in Figure 5E. In the taste buds, artificial sweeteners activate PLC-β_2_ via gustducin, and resultant elevation of [Ca^2+^]_c_ leads to an activation of TRPM5, which induces sodium entry and depolarizes the plasma membrane [15]. We therefore examined the expression of the channels belonging to the TRPM family. As shown in Figure 5F, TRPM5 was not detected in our experimental condition. Instead, TRPM4, which resembles TRPM5 in many respects, and TRPV2 were expressed.

### Effect of Sucralose on Activation of Protein Kinase C

**Figure 6:**
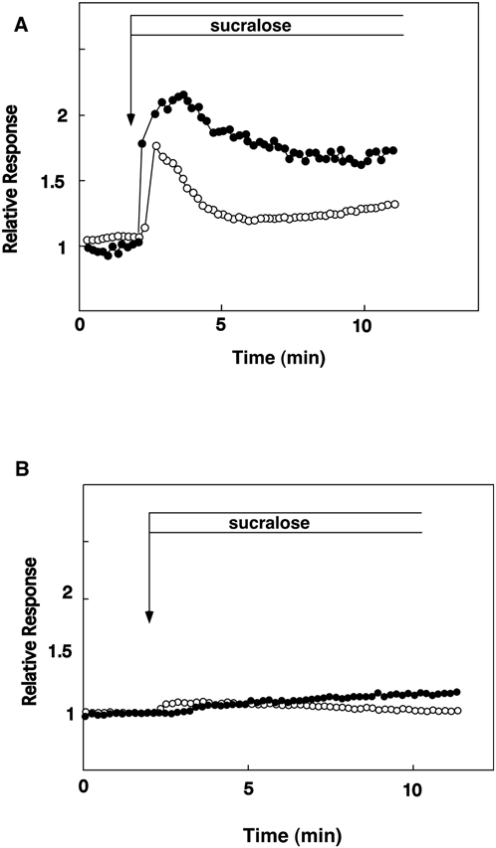
Effect of sucralose on PKC activation. (A) MIN6 cells expressing MARCKS-GFP were loaded with fura-2. Cells were stimulated with 50 mM sucralose and changes in [Ca^2+^]_c_ (○) and the amount of MARCKS-GFP in cytosol (•) were monitored. (B) MIN6 cells expressing MARCKS-GFP were loaded with fura-2. Cells were then incubated in Ca^2+^-free HBSS and stimulated with 50 mM sucralose and changes in [Ca^2+^]_c_ (○) and amount of MARCKS-GFP in cytosol (•) were measured.

To determine whether or not sucralose also activates PKC, we monitored translocation of a PKC substrate MARCKS. As reported previously, unphosphorylated MARCKS is localized in the plasma membrane and is released into cytosol when phosphorylated by PKC [16]. We therefore monitored the amount of cytosolic MARCKS by measuring the MARCKS-GFP fluorescence in cytosol. As shown in Figure 6A, addition of sucralose increased the MARCKS-GFP fluorescence in cytosol. The effect of sucralose was rapid and sustained for at least 10 min. In the absence of extracellular calcium, the effect of sucralose on cytosolic MARCKS was nearly completely inhibited (Figure 6B). A similar result was obtained by adding nifedipine (data not shown).

### Expression of the Sweet Taste Receptor in Mouse Islets

**Figure 7:**
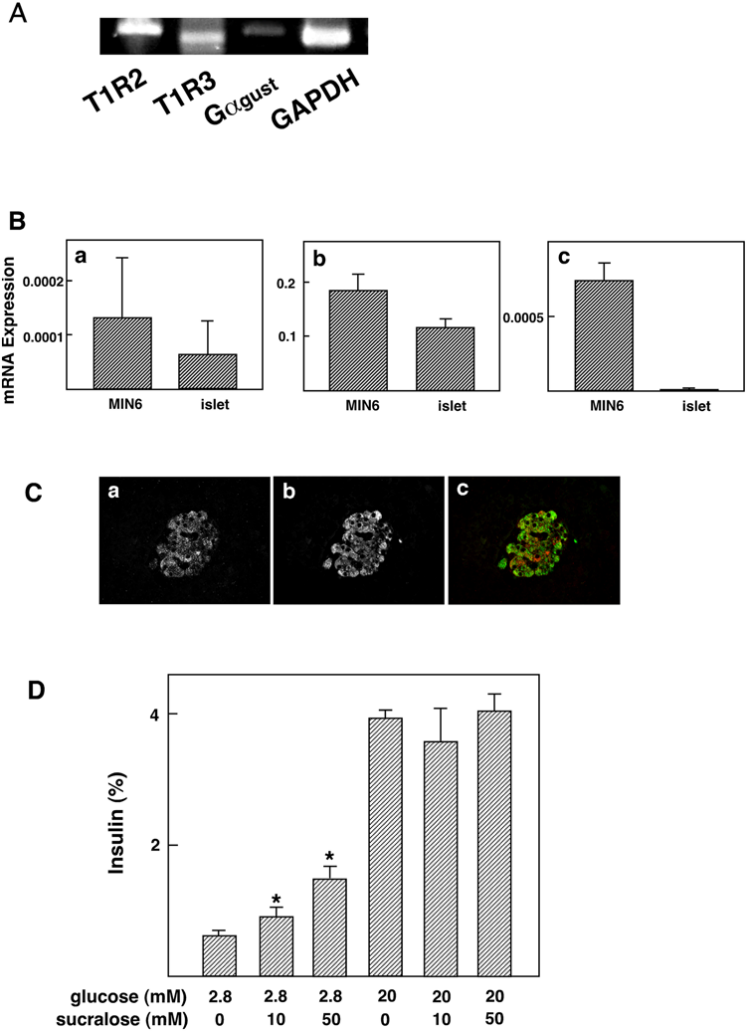
Expression of the sweet receptor in islets. (A) Expression of mRNA in Mouse Islets. mRNA was extracted from mouse islets and the expression of T1R2, T1R3, and Gα_gust_ was measured by RT-PCR. (B) Comparison of the Expression of T1Rs and Gustducin in Islets and MIN6 cells. mRNA levels for T1R2 (a), T1R3 (b) and Gα_gust_ (c) were measured by quantitative PCR in islets and MIN6 cells and expressed as relative to β actin. (C) Expression of T1R3 in Islets. Pancreatic slices were stained with anti-T1R3 (a) and anti-insulin (b) antibodies. c: merge. (D) Effects of Artificial Sweeteners on Insulin secretion from Islets. Islets were incubated for 60 min with various concentrations of sucralose in the presence of 2.8 and 20 mM glucose. Values are the mean±S.E. for four experiments. *: p<0.05 vs control.

We then examined the expression of the sweet taste receptor in mouse islets. As shown in Figure 7A, mRNA for T1R2 and T1R3 was detected in mouse islets. In addition, mRNA for gustducin was also detected in this condition. Figure 7B shows the comparison of the expression of T1Rs in islets and MiN6 cells. The expression levels of mRNA for T1Rs and gustducin were lower in islets compared to MIN6 cells. As shown in Figure 7C, immunoreactivity of T1R3 was found in insulin-producing β-cells. Immunoreactive T1R2 was not detected. To test the function of the sweet taste receptor, mouse islets were incubated with an artificial sweetener, sucralose. As depicted in Figure 7D, sucralose induced insulin secretion from mouse islets.

## Discussion

In the present study, we showed that mRNA and protein of the sweet taste receptor are expressed in pancreatic β-cells and MIN6 insulinoma cells. Also, gustducin, a trimeric GTP-binding protein coupling the sweet taste receptor and phospholipase C-β_2_ in the taste buds, is also expressed in β-cells and MIN6 cells. Thus, components of the sweet taste receptor signaling system are present in β-cells. These results are in agreement with a recent report by Reimann, et al. that mRNA for T1Rs is expressed in islets [17]. The sweet taste receptor system in β-cells is functional since several artificial sweeteners, which are known to be sweet taste receptor agonists in the taste buds, are able to stimulate insulin secretion from mouse islets as well as MIN6 cells. Hence, in addition to enteroendocrine cells [8], a functional sweet taste receptor system is expressed in pancreatic β-cells. Malaisse, et al. [18] reported that artificial sweeteners such as sodium saccharin, sodium cyclamate and acesulfame-K increased insulin secretion. These compounds also display a bitter taste. They postulated that these agents acted on the bitter taste receptor. In this regard, bitter-tasting substance denatonium stimulates insulin secretion [19]. This compound acts directly on Kir6.2 and blocks the ATP-sensitive potassium channel [19]. Given that sucralose, which does not display a bitter taste, stimulates insulin secretion, at least some of the effects of sodium saccharin and acesulfame-K are mediated by the sweet taste receptor.

In MIN6 cells, sucralose stimulated insulin secretion in the presence of 3 mM glucose but not in the presence of 25 mM glucose. In contrast, succharin and acesulfame-K augmented insulin secretion induced by 25 mM glucose. This could be due to the fact that, unlike sucralose, high concentration of acesulfame-K and succharin may cause depolarization.

We investigated the intracellular signaling system activated by the sweet taste receptor in MIN6 cells. We monitored changes in [Ca^2+^]_c_ and [cAMP]_c_ in living MIN6 cells using a fluorescent indicator fura-2 and fluorescent proteins GFP, CFP and YFP. The results show that sucralose increased both [Ca^2+^]_c_ and [cAMP]_c_, and in addition, induced phosphorylation of PKC substrate MARCKS. Indeed, sucralose increased [Ca^2+^]_c_ by inducing mobilization of calcium from an intracellular pool and also stimulation of calcium entry. Since mobilization of calcium was blocked by an inhibitor of the Ins-P_3_ receptor 2APB (13), it is likely that the sweet taste receptor agonist sucralose activates PLC in MIN6 cells. Nevertheless, the effects of sucralose on [Ca^2+^]_c_ and [cAMP]_c_ are slightly different from those of other calcium mobilizing agonists such as carbachol, which activates PLC via an activation of G_q_. For example, a muscarinic agonist carbachol activates PLC-β and increases [Ca^2+^]_c_ by causing calcium release via the Ins-P_3_ receptor and stimulation of calcium entry. Unlike sucralose, however, carbachol instead reduced [cAMP]_c_. This may have been due to an activation of G_i_ by the muscarinic receptor. In contrast, sucralose significantly increased [cAMP]_c_. These results suggest that the sweet taste receptor activated PLC-β by a different mechanism compared to carbachol. Consistent with this notion, sucralose-induced effects on [Ca^2+^]_c_ and [cAMP]_c_ were not affected by a G_q/11_ inhibitor YM254890, while YM254890 completely abolished the carbachol effects. Consequently, it is likely that sucralose may activate PLC by a mechanism independent of G_q_ activation. In the taste buds, the sweet taste receptor is coupled to gustducin, which is also expressed in MIN6 cells. We therefore speculate that the sweet taste receptor is also coupled to gustducin in MIN6 cells. In this regard, [Ca^2+^]_c_. response induced by sucralose was inhibited by gurmarin. Gurmarin is an inhibitor of the sweet taste receptor and is thought to affect the activation of gustducin by T1R2 + T1R3 in the tast buds [12]. Collectively, it is conceivable that at least some of the signals generated by the sweet taste receptor in MIN6 cells are mediated by gustducin. With regard to an increase in cAMP induced by sucralose, the present results show that most of the cAMP signal was inhibited by reduction of calcium entry. Calcium-dependent activation of adenylate cyclase and/or inhibition of phosphodiesterase may have been involved. However, some increase was observed in [cAMP]_c_ even in the presence of 2APB, which completely blocked sucralose-induced elevation of [Ca^2+^]_c_. This raises a possibility that activation of the sweet taste receptor also increases [cAMP]_c_ by a calcium-independent mechanism possibly through G_s_.

The results shown in Figure 5A indicate that sucralose-mediated elevation of [Ca^2+^]_c_ was greatly dependent on extracellular calcium. Also, nifedipine markedly reduced elevation of [Ca^2+^]_c_ induced by sucralose. It is likely that sucralose eventually activated the voltage-dependent calcium channel and augmented calcium entry. In this regard, removal of extracellular sodium blocked calcium entry induced by sucralose. Sucralose may activate sodium-permeable cation channel and depolarizes the plasma membrane, which leads to the activation of the voltage-dependent calcium channel. In the taste buds of the tongue, the sweet taste receptor activates TRPM5 and induces sodium entry, which is a prerequisite for sweet sensation [15]. As shown in Figure 5F, TRPM5 is not expressed in MIN6 cells. It seems likely that other members of the TRP family may be involved in the action of sucralose. In this regard, TRPM2 and TRPM4 are expressed in MIN6 cells. TRPM4 is a sodium-permeable cation channel activated by Ca^2+^ and is involved in the regulation of insulin secretion (20). TRPM2 is a calcium-permeable channel and is involved in insulin secretion [21]. Further studies are necessary to determine the involvement of these channels in the action of sucralose.

The present results show that the sweet taste receptor is expressed in β-cells and is functionally operative. In addition to the taste buds in the tongue [1]–[4] and enteroendocrine cells [8], pancreatic β-cells express the sugar-sensing receptor system. At present, the physiological role of the sweet taste receptor in β-cells is not clear. The obvious candidate ligand for this receptor is glucose. However, the patterns of the changes in intracellular signals generated by high concentration of glucose (Figure 3C) and sucralose (Figure 3A) are quite different. Furthermore, the effect of glucose on [Ca^2+^]_c_ is blocked by diazoxide, whereas the effect of sucralose is not affected by diazoxide. Most of the signals generated by high concentration of glucose may be independent of the sweet taste receptor. Then the question is what is a natural ligand for the taste receptor expressed in β-cells? Given that the sweet taste receptor is able to be activated by a variety of compounds with different structures, it is tempting to speculate that an endogenous ligand(s) for the sweet taste receptor other than glucose may exist, and it modifies the β-cell functions through activation of the sweet taste receptor. Further studies are required to address this issue. From a practical point of view, the present study raises an interesting possibility that agonists for the sweet taste receptor may be good therapeutic candidates to augment insulin secretion. For this purpose, the putative compounds or artificial sweeteners should be absorbed efficiently as an intact molecule and should have access to β-cells via circulation. Since artificial sweeteners are not fuel, they provide insulin secretagogues without affecting the metabolism. Given that sweet taste receptor agonists increase calcium and especially cAMP in β-cells, they can be useful stimulators of insulin secretion. In this regard, it should be mentioned that sucralose is an artificial sweetener and used as a noncaloric sweeteners to reduce the intake of sugars. It is shown that the compound does not affect the glycemic control in diabetic patients [22], [23]. However, since sucralose increases insulin secretion in a dose-dependent manner, high dose of the compound may aggravate hypoglycemia.

## Materials and Methods

### Cell Culture

MIN6 cells (passages 16–24) [24] were grown in Dulbecco's modified Eagle's medium containing high glucose (Invitrogen, Carlsbad, CA) and 15% fetal bovine serum and cultured in a humidified incubator 95% air and 5% CO_2_ at 37°C.

### Isolation of Total RNA and Quantitative RT–PCR

Total RNA of MIN6 cells was isolated by using the TRIZOL Reagent (Invitrogen), and that of mice islets was isolated by using the RNeasy Mini Kit (Qiagen, Valencia, CA). Then these total RNAs were used for cDNA synthesis using Superscript II reverse transcriptase (Invitrogen), random primer (Takara Bio, Inc., Shiga, Japan) and oligo (dT)^12–18^ (Invitrogen). Quantitative RT-PCR was performed with 7500 Fast Real-Time PCR System (Applied Biosystems, Foster City, CA). The PCR reaction mixture consisted of first-strand cDNA template, SYBR GREEN PCR Master Mix (Applied Biosystems) and primer sets. For T1R2, the forward primer was 5′-GTCCGCTGCACCAAGCA-3′ and the reverse primer was 5′-GTTCGTCGAAGAAGAGCTGGTT-3′. For T1R3, the forward primer was 5′-TCAGAGCTTGCCCTCATTACAG-3′ and the reverse primer was 5′-TGTGCGGAAGAAGGATGGA-3′. For Gα_gust_, the forward primer was 5′-TGCATTATATTTTGCGCAGC-3′ and the reverse primer was 5′- AGAACAATGGAGGTGGTTGC-3′. For β-actin, the forward primer was 5′-AGGATGCAGAAGGAGATTACTG-3′ and the reverse primer was 5′- GCRGATCCACATCTGCTGGAA-3′. In all cases, expression was corrected with that of β-actin measured on the same sample in parallel on the same plate.

### RT–PCR

Total RNA isolated from MIN6 cells and mice islets was used for cDNA synthesis using Superscript III reverse transcriptase (Invitrogen) and oligo (dT)^12–18^. The cDNA was used for PCR template using Taq DNA Polymerase, recombinant (Invitrogen) and the following primer sets: For T1R2 mRNA, the forward primer was 5′-CACGATGAGGAAGAGCTAGTC-3′ and the reverse primer was 5′-CACCCAAGGGAAATTTATTCATA-3′. For T1R3 mRNA, the forward primer was 5′-AAATGTACTGGCCAGGCAAC-3′ and the reverse primer was 5′-GTGAGCCATTGGTTGTTGTG-3′. For Gα_gust_ mRNA, the forward primer was 5′-CTGGTATCATTGAAACTCAATTCTCC-3′ and the reverse primer was 5′-GAGCCCACAGTCTTTGAGGTTC-3′. For control GAPDH mRNA, the forward primer was 5′-ATGGGAAGCTGGTCATCAAC-3′ and the reverse primer was 5′-GGATGCAGGGATGATGTTCT-3′. For TRPM1 mRNA, the forward primer was 5′-ATCCGAGTCTCCTACGACACCAAG -3′ and reverse primer was 5′- TCTTTCAAGGCATCCCCCAC-3′. For TRPM2 mRNA, the forward primer was 5′-CCAATCTCCGACGAAGCAATAG-3′ and reverse primer was 5′- GCTCAGGTCTGTGAAAACGATGTC-3′. For TRPM3 mRNA, the forward primer was 5′-CGTGGGGGACCGTTTATTTTC-3′ and reverse primer was 5′- GAAGCACAGAGATACTGGGAGTGAG-3′. For TRPM4 mRNA, the forward primer was 5′-GCTTTCGTGCCCCAAACTTG-3′ and reverse primer was 5′- TTCTGCTGAGCCACATAGGACTC-3′. For TRPM5 mRNA, the forward primer was 5′-GGAAGAAGCGAGGCAAGTTTG-3′ and reverse primer was 5′- GAAGTTGACGGTGAAGGATTCG-3′. For TRPM6 mRNA, the forward primer was 5′-GAAGCACACAGTGAAAAGCCCTAC-3′ and reverse primer was 5′- GGTTCTCAATGACTCCCCAAGG-3′. For TRPM7 mRNA, the forward primer was 5′-TCCAGGATGTCAGATTTGTCAGC-3′ and reverse primer was 5′- CTGTAGGAATGAGAACCCCCTTG-3′. For TRPM8 mRNA, the forward primer was 5′-ATGAGGAGCCGCAGAAATGG-3′ and reverse primer was 5′- CCAGGTTGGGTGTTTTGAGGTG-3′.

### Transfection

Cells were transiently transfected with 5.4 µg of plasmid encoding Epac1-camp [25] or MARCKS-GFP [26] using Lipofectamine 2000 transfection reagent (Invitrogen) according to the manufacturer's instructions. Transfection efficiencies ranged between 70 and 80%.

### Imaging of Epac1-camps, MARCKS-GFP, and Fura-2

Transfected MIN6 cells were placed on a 35 mm glass bottom culture dish. Before the measurements, growth medium was removed and replaced with Hanks' balanced salt solution (HBSS) containing 1.3 mM CaCl_2_, 5.4 mM KCl, 0.44 mM KH_2_PO_4_, 0.5 mM MgCl_2_, 0.38 mM MgSO_4_, 138 mM NaCl, 0.34 mM Na_2_HPO_4_, 3.0 mM D-glucose and HEPES-NaOH (pH 7.4). To remove extracellular sodium, sodium was replaced by choline chloride. For imaging, cells were visualized with a 40 Uapo/340 objective lens (Olympus, Tokyo, Japan). The Epac1-camps excitation wavelength was 440 nm and attenuated by 6% using neutral density filters. For dual emission ratio imaging for enhanced cyan fluorescent protein (ECFP) and enhanced yellow fluorescent protein (EYFP), we used AQUACOSMOS/ASHURA, a 3CCD based fluorescence energy transfer imaging system (Hamamatsu Photonics, Hamamatsu, Japan). MARCKS-GFP [26] fluorescence was obtained by excitation at 488 nm. Images (200–300 ms exposure) were captured with a 12-bit C7780-22 ORCA3CCD camera (Hamamatsu Photonics) at 10 s intervals. Epac1-camps FRET was decreased with increasing cAMP concentration: an increase in the FRET emission ratio is indicative of an increase in cytoplasmic cAMP concentration ([cAMP]_c_) [25]. Fluorescence intensity in the cytosol of the cells expressing MARCKS-GFP was also measured. These values (F) were normalized to each initial value (F_0_) so that the relative fluorescence change was referred to as F/F_0_.

For simultaneous measurements of intracellular Ca^2+^ and cAMP levels or intracellular Ca^2+^ and MARCKS-GFP translocation, we used fura-2 to detect cytoplasmic Ca^2+^ concentration ([Ca^2+^]_c_) [27]. Spectral separation of Epac1-camps and fura-2 or MARCKS-GFP and fura-2 excitation and emission permits simultaneous imaging of [cAMP]_c_ and [Ca^2+^]_c_ or MARCKS-GFP and [Ca^2+^]_c_ in a single cell. For concurrent [cAMP]_c_ and [Ca^2+^]_c_ or MARCKS-GFP and [Ca^2+^]_c_ measurements, MIN6 cells transiently transfected with Epac1-camps or MARCKS-GFP were loaded with 1.5 µM fura-2/AM (Dojindo, Kamimashiki, Japan) for 20 min at room temperature. Excitation filters of 340- and 380-nm and a 530-nm emission filter were used for fura-2 dual excitation ratio imaging. A U-MGFPHQ fluorescence mirror unit (Olympus) was used for MARCKS-GFP. Imaging data acquisition and analysis were accomplished using Aquacosmos 2.6 software (Hamamatsu Photonics). Excitation filters of 340- and 380-nm were used for fura-2 dual excitation and these images were obtained by yellow channel of the 3CCD camera. The backgrounds of the emission intensities were subtracted. Data are expressed as the ratio of FRET cyan channel and yellow channel intensity, the ratio of fura-2 340 and 380 nm excitation (340/380 ratio) and the ratio of MARCKS-GFP cytosolic fluorescence intensity and initial intensity (F/F_0_). In addition, data were normalized to the average base-line values of the 485/535 and 340/380 nm ratio (485/535 and 340/380 nm relative ratio, respectively) to facilitate comparisons between responses in different cells. Data are expressed as means±S.E.

### Animals

C57BL/6J mice were purchased from CLEAR Japan (Tokyo, Japan). They were kept in an experimental animal facility controlled at 23°C room temperature with a 12-hr light and dark cycle, and with free access to standard chow and water.

The animal experiment was conducted according to the guidelines for animal care issued by The Animal Experiment and Ethic Committee, Gunma University, School of Medicine.

### Histology and Immunohistochemistry

Male C57BL/6J mice were anesthetized with ether. Pancreata were excised and fixed in Bouin's fixative at 4°C overnight. The pancreata were embedded in paraffin, and serial holizontal sections of 5 µm were cut, deparaffinized, and immunofluorescence was performed as described previously [28]. Antigen retrieval consisted of treatment of paraffin sections three times for 5 min each with 0.01M citrate buffer in a microwave oven, followed by a 20 min cool-down period before proceeding to the next step. For immunohistochemistry, sections were treated with 1% bovine serum albumin (BSA) in phosphate-buffered saline (PBS) for 30 min to block nonspecific antibody binding. The following primary antibodies were diluted with PBS containing 1% BSA and applied to the sections: rabbit polyclonal anti-G Protein-Coupled Receptor TAS1R2 antibody (1∶100, LifeSpan Biosciences, Seattle, WA), rabbit polyclonal anti-G Protein-Coupled Receptor TAS1R3 antibody (1∶100, MBL International Corporation, Woburn, MA), rabbit polyclonal anti-glucagon antibody (1∶500; DAKO, Carpenteria, CA), and guinea pig polyclonal anti-insulin antibody (1∶1,000; DAKO). For enzymatic detection of the bound primary antibodies, Alexa Fluor 488-conjugated anti-rabbit IgG, Alexa Fluor 568-conjugated anti-guinea pig IgG, and Alexa Fluor 568-conjugated anti-rabbit IgG (Molecular Probes, Oregon) were used at 1∶200 dilution (PBS with 1% BSA). Nuclear counterstaining was achieved by 4′, 6-Diamidino-2-phenylindole-HCl (DAPI) (PIERCE, Rockford, IL). Immunocytochemical images were taken on a confocal laser scanning biological microscope FV1000-D (OLYMPUS, Tokyo, Japan).

### Immunofluoresence Microscopy of Cultured Cells

MIN6 cells grown on the micro cover glass (MATSNAMI Glass., ltd., Tokyo, Japan) were fixed with 3% paraformaldehyde over night at 4°C. Fixed cells were incubated in Glycine/PBS (containing 50 mM glycine ) for 3 min and subsequently incubated in PBS-T (containing 0.1% v/v Triton X-100 ) for 5 min at room temperature. Cells were then incubated overnight at 4°C with either T1R2 antibody or T1R3 antibody, each used at a dilution of 1∶800. After 3 times washing with PBS, cells were incubated with the Alexa 488-conjugated secondary antibody (dilution; 1∶1000, In Vitrogen) and DAPI (dilution; 1∶1000) for 1 hour at room temperature. All of the presented images were captured using confocal laser scanning biological microscope FV1000-D (OLYMPUS).

### Evaluation of Insulin Secretion

MIN6 cells were seeded into 24-well plates and incubated for 48–72 h before measurement of insulin secretion. Cells were preincubated for 1h in Krebs-Ringer-HEPES (KRH) buffer (125 mM NaCl, 5.9 mM KCl, 1.28 mM CaCl_2_, 5.0 mM NaHCO_3_, 25 mM HEPES/NaOH, and 0.1% (w/v) bovine serum albumin) in a 37°C humidified incubator [29]. The cells were then incubated in the same buffer containing 2.8, 8.3 or 25 mM glucose in the presence and absence of artificial sweeteners. The supernatant of the cells was collected and centrifuged at 300 g for 10 min to remove cell debris. The insulin concentration in the supernatant was measured by radioimmunoassay (Eiken Chemical, Tokyo, Japan) according to the manufacturer's protocol. The insulin secretion data were normalized by insulin content. Mouse islets were isolated by collagenase digestion [30] and were preincubated for 1 h in KRH buffer. Then the islets were incubated for 1 h in KRH buffer containing various compounds. The supernatant was collected and the insulin concentration was measured.
